# Hydrogen-bonding Interactions between Apigenin and Ethanol/Water: A Theoretical Study

**DOI:** 10.1038/srep34647

**Published:** 2016-10-04

**Authors:** Yan-Zhen Zheng, Yu Zhou, Qin Liang, Da-Fu Chen, Rui Guo, Rong-Cai Lai

**Affiliations:** 1 College of Bee Science, Fujian Agriculture and Forestry University, Fuzhou 350002, P. R. China; 2 Key Laboratory of Bioorganic Phosphorous Chemistry and Chemical Biology (Ministry of Education), Department of Chemistry, Tsinghua University, Beijing 100084, P. R. China

## Abstract

In this work, hydrogen-bonding interactions between apigenin and water/ethanol were investigated from a theoretical perspective using quantum chemical calculations. Two conformations of apigenin molecule were considered in this work. The following results were found. (1) For apigenin monomer, the molecular structure is non-planar, and all of the hydrogen and oxygen atoms can be hydrogen-bonding sites. (2) Eight and seven optimized geometries are obtained for apigenin (I)–H_2_O/CH_3_CH_2_OH and apigenin (II)–H_2_O/CH_3_CH_2_OH complexes, respectively. In apigenin, excluding the aromatic hydrogen atoms in the phenyl substituent, all other hydrogen atoms and the oxygen atoms form hydrogen-bonds with H_2_O and CH_3_CH_2_OH. (3) In apigenin–H_2_O/CH_3_CH_2_OH complexes, the electron density and the E(2) in the related localized anti-bonding orbital are increased upon hydrogen-bond formation. These are the cause of the elongation and red-shift of the X−H bond. The sum of the charge change transfers from the hydrogen-bond acceptor to donor. The stronger interaction makes the charge change more intense than in the less stable structures. (4) Most of the hydrogen-bonds in the complexes are electrostatic in nature. However, the C4−O5···H, C9−O4···H and C13−O2···H hydrogen-bonds have some degree of covalent character. Furthermore, the hydroxyl groups of the apigenin molecule are the preferred hydrogen-bonding sites.

Propolis (or bee glue) has been used as a popular natural remedy in folk medicine for centuries all over the world^1^. Propolis is a strongly adhesive, resinous substance that honey bees collect from buds, barks and plant exudates and use in their beehives as a protective barrier against invaders and contamination^2^,^3^. It is reported that propolis possesses a broad array of biological properties, such as anti-septic, anti-mycotic, anti-viral, spasmolytic, anti-inflammatory and immunostimulatory activities^2^. Due to these characteristics, which can yield health benefits, propolis has attracted much attention in recent years as an important substance for use in foods and medicines to improve health and to prevent diseases.

Although the precise composition of raw propolis varies based on several factors, such as geographical zones and botanical origins, propolis is generally composed of 50% resin and balsam, 30% wax, 10% aromatic oil, 5% pollen and 5% various other substances^3^. The chemical composition of propolis is complicated. More than 300 compounds, including aromatic acids, esters, waxy acids, flavonoids and terpenoids have been isolated from raw propolis^2^. Among those constituents, flavonoids are one of the most important bioactive compounds and are reported to be responsible for some of the medicinal properties of propolis such as anti-microbial, anti-cancer, anti-oxidant and anti-viral activities^4^,^5^.

As raw propolis is a hard resinous product, it cannot be consumed in its natural form. Before used, raw propolis must be purified by solvent extraction to remove the inert materials (such as resin, balsam and beeswax) and to preserve the flavonoid fractions. Ethanol^6^,^7^ and water^8^,^9^ have been considered as good solvents to extract flavonoids from propolis. The anti-oxidant activity of ethanolic propolis from various geographical origins was studied, and different activities were found for each sample^6^. Aqueous propolis extracts also showed an effective antioxidant activity in several *in vitro* assays^8^,^9^. The bioactive properties of the aqueous and ethanolic extracts are attributed mainly to the high content of flavonoids in the mixture. Although, the extraction of flavonoids from propolis by water and ethanol under different conditions have been investigated^6^,^7^,^8^,^9^, few works have focused on the mechanism of the extraction process, the microstructure and the physical properties of the extracting solution. In liquids, intermolecular interactions, particularly hydrogen-bonding interactions, greatly influence the extraction progress and the physical properties of the solution^10^. The investigation of the hydrogen-bonding interactions in mixtures of flavonoids and water/ethanol is particularly important to understand the mechanism of the extraction process and the physical essence of the mixture. Quantum chemical calculations have been widely and effectively used to study the intermolecular interactions theoretically^11^,^12^,^13^,^14^,^15^,^16^,^17^,^18^,^19^,^20^,^21^. In this work, the hydrogen-bonding interactions between ethanol/water and flavonoids were investigated from a theoretical perspective. 5,7-Dihydroxy-2-(4-hydroxyphenyl)-phenylchromen-4-one (apigenin) was selected as the representative flavonoid. Density functional theory (DFT) and MP2 methods were used to reveal the hydrogen-bonding interactions from a theoretical viewpoint.

## Results and Discussion

### Apigenin monomer geometry analysis

**Figure 1:**
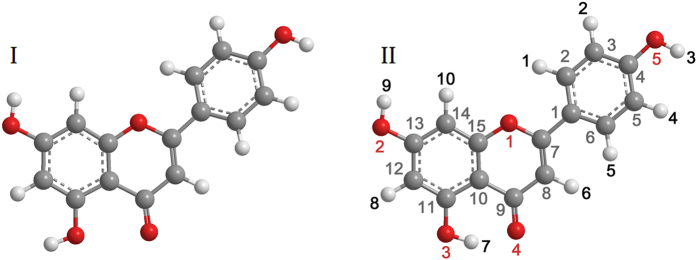
Conformations and atom numberings for all of the atoms of apigenin (I) and apigenin (II). The labels of oxygen, hydrogen and carbon atoms are in red, blue and gray colors, respectively.

Two conformations of the apigenin molecule, labeled apigenin (I) and apigenin (II), were considered in this work. The conformation and atom numbering for the apigenin monomer are shown in Fig. 1. The labels of oxygen, hydrogen and carbon atoms are in red, blue and gray colors, respectively. In apigenin (II), the binding distance between H7 and O4 is approximately 1.7 Å, which is less than the sum of the van der Waals atomic radii of hydrogen and oxygen^22^. This result indicates that there is an intramolecular hydrogen-bond between H7 and O4 in apigenin (II).

The optimized bond lengths, bond angles and dihedral angles for apigenin (I) and apigenin (II) calculated using the B3LYP/6−31 + + G(d, p), M062X/6−31 + + G(d, p) and MP2/6−31 + + G(d, p) methods are listed in Tables S1 and S2. As shown in Tables S1 and S2, the C9−O4 bond shows typical double bond characteristics, whereas the other C−O bonds exhibit single bond characteristics. Most values of the C−C bond lengths are approximately 1.400 Å, close to the normal C−C single bond length in both methods. The C1−C7 bond connecting the phenyl ring to the chromone part is approximately 1.470 Å based on calculations using the three abovementioned methods for apigenin (I) and apigenin (II). Because this bond plays a bridging role between the chromone part and the phenyl ring of apigenin, the conjugation of the phenyl ring and chromone is suggested. C8−C9 and C9−C10 bond lengths are approximately 1.460 Å for the two conformations. The longer bond length mainly due to the electronegativity of the keto substituent in the heterocyclic ring.

The bond angles of the aromatic ring are approximately 120°. However, the C8−C9−C10 bond angle in the heterocyclic ring deviates from 120°, mainly due to the conjugation across the heterocyclic ring and the keto group.

For the two conformations of the apigenin molecule, the C9−C10−C15−C14 and C13−C14−C15−C10 dihedral angles are very close to 180° and 0°, respectively. These results indicate that the chromone part is almost planar in orientation. The dihedral angles between the chromone and the phenyl group (C2−C1−C7−C8 and C2−C1−C7−O1) are approximately 160/156/154° and −19/−23/−25° as calculated using the B3 LYP, M062X and MP2 methods, respectively, which indicates that the phenyl substituent is out of the plane with the chromone part. Therefore, the molecular structure of apigenin is non-planar.

For apigenin (II), the optimized results are very close to the data from the literature^23^, which further verify the calculated results of this work.

### Charge analysis of apigenin monomer

**Figure 2:**
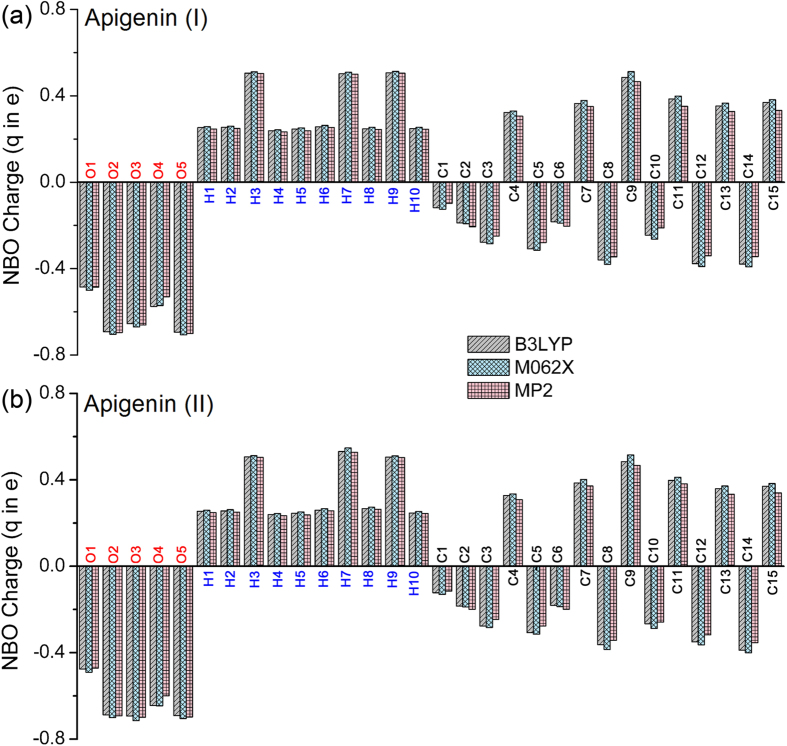
Histogram of the calculated NBO charges for apigenin.

The calculation of the effective atomic charge plays an important role as an indicator of the possible interaction sites of the apigenin monomer. Figure 2 presents the charge distributions of the optimized apigenin monomer examined using the NBO (natural bond orbital) analysis.

In the apigenin monomer, all of the oxygen atoms have negative charges, and the oxygen atoms (O2, O3 and O5) of the hydroxyl groups have the most negative charges, followed by the carbonyl oxygen atom (O4) and the ether oxygen atom (O1). All of the hydrogen atoms have positive charges, and the hydrogen atoms on the hydroxyl groups (H3, H7 and H9) have more positive charges than the other hydrogen atoms due to their bond with the more electronegative oxygen atom (O2, O3 and O5). These results indicate that all of the oxygen and hydrogen atoms in apigenin can be hydrogen-bond acceptors and donors and that the oxygen and hydrogen atoms in the hydroxyl groups may be the preferred interaction sites over the other oxygen and hydrogen atoms.

The carbon atom C9 has the highest positive charge compared with the other ring carbon atoms, as shown in the histogram, owing to the electronegative keto group.

### Optimized geometries of apigenin–H_2_O/CH_3_CH_2_OH complexes

The hydrogen-bonding interactions in liquids play important roles; thus, in this work, the hydrogen-bonds in apigenin–H_2_O/CH_3_CH_2_OH complexes were examined in detail. The definition of hydrogen-bond according to IUPAC is that a hydrogen-bond is an attractive interaction between a hydrogen atom from a molecule or a molecular fragment X–H, in which X is more electronegative than H, and an atom or a group of atoms in the same or a different molecule, in which there is evidence of bond formation^24^. The hydrogen-bond is often expressed as X−H···Y. Based on the definition of a hydrogen-bond, in this work, the sum of van der Waals atomic radii of hydrogen and oxygen (2.5 Å) was used as a critical value for judging the existence of a hydrogen-bond^22^. The optimized geometries were performed using the B3 LYP/6−31 + + G(d, p) method. The above section states that all of the hydrogen atoms and oxygen atoms can serve as an interaction site. Therefore, in the optimization process, H_2_O and CH_3_CH_2_OH were placed around all of the hydrogen atoms and the oxygen atoms to examine all of the possible apigenin−H_2_O/CH_3_CH_2_OH geometries.

**Figure 3:**
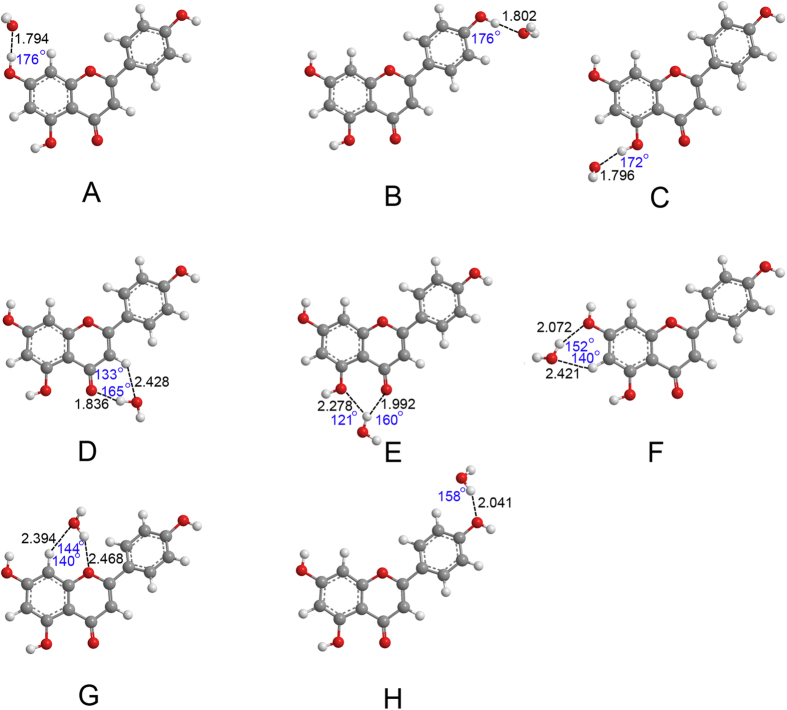
Optimized geometries for the apigenin (I)–H_2_O complex using the B3LYP/6−31 + + G(d, p) method. Hydrogen-bonds are denoted by dashed lines, and the corresponding H···O distances (Å) and bond angles (°) are labeled in black and blue colors, respectively.

**Figure 4:**
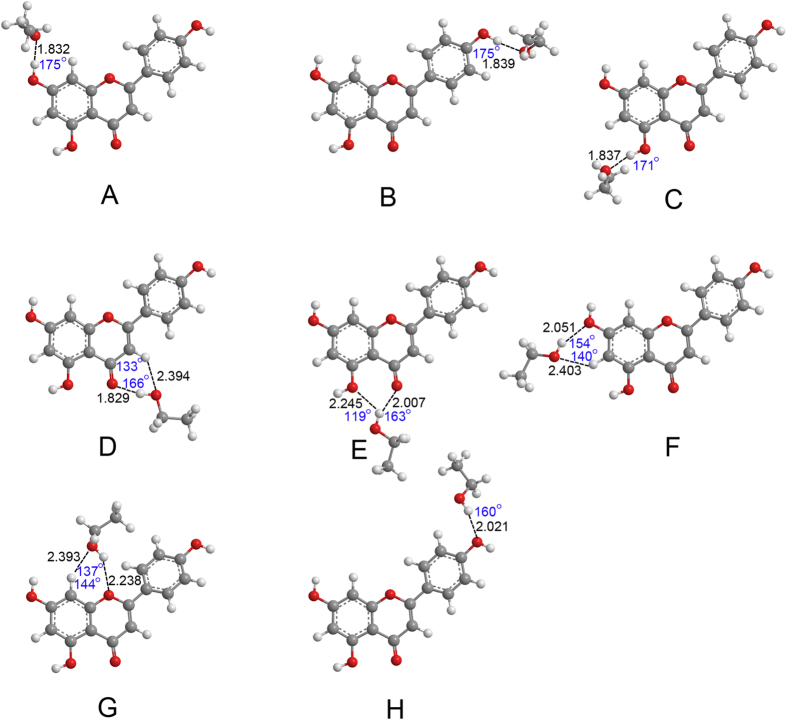
Optimized geometries for the apigenin (I)–CH_3_CH_2_OH complex using the B3LYP/6−31 + + G(d, p) method. Hydrogen-bonds are denoted by dashed lines, and the corresponding H···O distances (Å) and bond angles (°) are labeled in black and blue colors, respectively.

**Figure 5:**
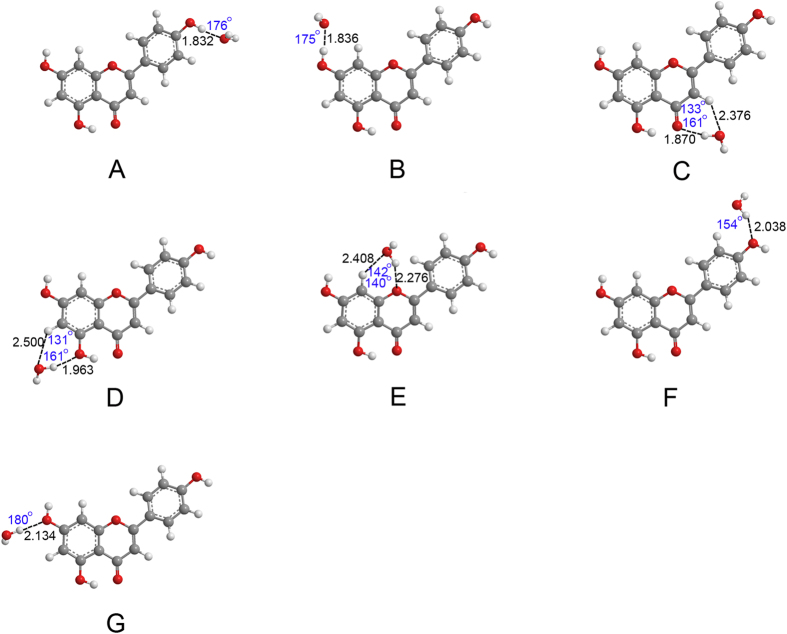
Optimized geometries for the apigenin (II)–H_2_O complex using the B3LYP/6−31 + + G(d, p) method. Hydrogen-bonds are denoted by dashed lines, and the corresponding H···O distances (Å) and bond angles (°) are labeled in black and blue colors, respectively.

**Figure 6:**
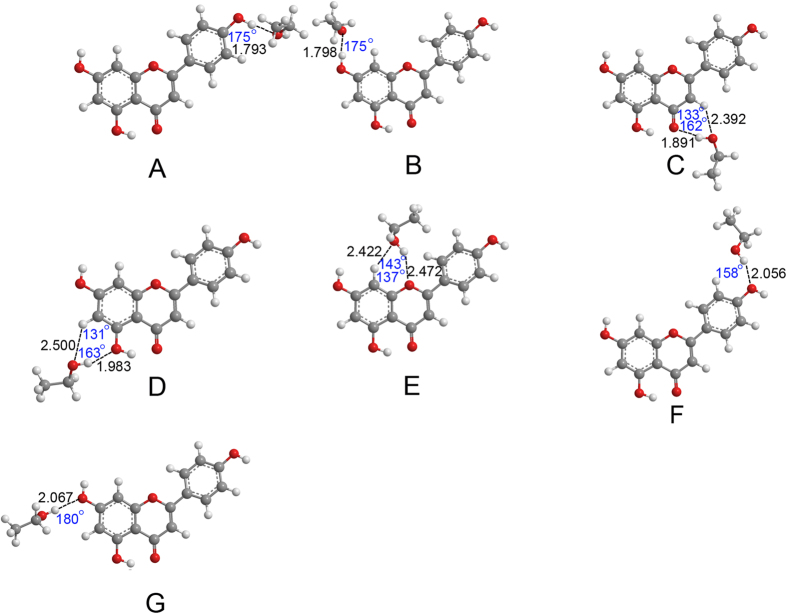
Optimized geometries for the apigenin (II)–CH_3_CH_2_OH complex using the B3LYP/6−31 + + G(d, p) method. Hydrogen-bonds are denoted by dashed lines, and the corresponding H···O distances (Å) and bond angles (°) are labeled in black and blue colors, respectively.

The optimized geometries of apigenin–H_2_O and apigenin–CH_3_CH_2_OH complexes are shown in Figs 3, 4, 5 and 6. Only the most stable optimized geometries are presented in this paper. As shown in the figures, for apigenin (I), both apigenin (I)–H_2_O and apigenin (I)–CH_3_CH_2_OH complexes have eight interaction structures. For apigenin (II), both apigenin (II)–H_2_O and apigenin (II)–CH_3_CH_2_OH complexes have seven interaction structures. For simplicity, the structures that have similar interaction sites are labeled with the same letters. The binding distances are in the 1.794–2.500 Å range and for the thirty structures are less than 2.5 Å. These results show that all of these structures are stable hydrogen-bonded complexes. For the structure with only one hydrogen bond, the X–H···Y bond angles are larger than 154°. For the structures possessing two hydrogen-bonds, the X–H···Y bond angles are between 119° and 166°. These values are within the X–H···Y bond angles range that is characteristic of hydrogen-bond complexes, thus indicating the formation of hydrogen-bond interactions in these complexes.

The NBO results in Section 3.2 show that all the hydrogen and oxygen atoms in apigenin can be interaction sites, however, as shown in Figs 3, 4, 5 and 6, not all of the hydrogen atoms have formed hydrogen-bonds with H_2_O and CH_3_CH_2_OH. Except for the H7 in apigenin (II), which has formed an intramolecular hydrogen-bond, all of the hydrogen atoms in the hydroxyl groups of apigenin interact with H_2_O and CH_3_CH_2_OH due to their large positive electron density compared with the other hydrogen atoms, as illustrated in Figs 3, 4, 5 and 6. Although, the electron densities of all the aromatic hydrogen atoms are very close, only the hydrogen atoms in the chromone part formed hydrogen-bonds with H_2_O and CH_3_CH_2_OH (structure D, F and G for apigenin (I), structure C, D, E for apigenin (II)) due to the synergistic effect of the other groups of the same structure. It is evident that in these structures (structure D, F and G for apigenin (I), structure C, D, E for apigenin (II)), the hydrogen atoms and oxygen atoms in H_2_O and CH_3_CH_2_OH interact with apigenin simultaneously, forming two hydrogen-bonds with a stable six-atom ring. An interesting phenomenon is that the hydrogen atom in the hydroxyl groups of H_2_O and CH_3_CH_2_OH formed dihydrogen-bonds with hydrogen atoms in the keto group and the hydroxyl group in apigenin, as illustrated in structure E of apigenin (I).

The above results indicate that H_2_O and CH_3_CH_2_OH formed different hydrogen-bonds with apigenin.

### Interaction energies of apigenin–H_2_O/CH_3_CH_2_OH complexes

**Figure 7:**
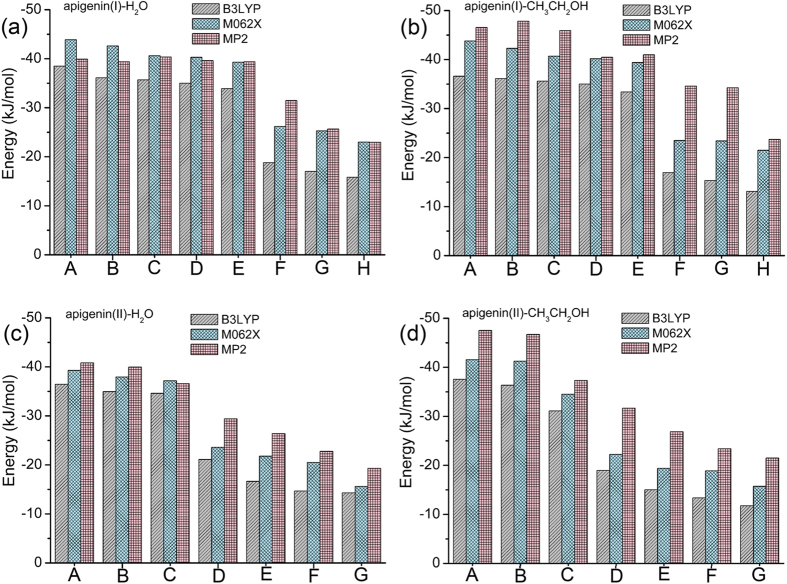
Histogram for the interaction energies of apigenin–H_2_O/CH_3_CH_2_OH complexes.

The interaction energy is a most convincing measure of the strength of non-covalent interactions. The relative stability of different conformers is in accordance with the calculated interaction energies. For a stable interaction complex, the value of the interaction energy (*Δ*E) is often negative. The larger the absolute value of *Δ*E the stronger the strength of the corresponding hydrogen-bond. In this work, the *Δ*E values of different geometries are drawn as histograms in Fig. 7. As shown in the figure, the absolute value of *Δ*E decreases from structure A to H. H_2_O and CH_3_CH_2_OH form strong hydrogen-bonds with the hydrogen atoms in the hydroxyl groups (structures A, B, and C for apigenin (I), structures A and B for apigenin (II)) and the oxygen atoms of the carbonyl groups (structures D and E for apigenin (I), structure C for apigenin (II)) of apigenin. For apigenin (I)−H_2_O/CH_3_CH_2_OH, the energies of the conformers A to E are close to each other and significantly higher than those of F to H. For apigenin (II)−H_2_O/CH_3_CH_2_OH, the energies of conformers A to C are close to each other and significantly higher than those of D to G. Because the atomic charges of the hydrogen atoms in the hydroxyl group of the apigenin monomer are very close, the interaction energies of the corresponding apigenin–H_2_O/CH_3_CH_2_OH complex are very similar (structures A, B, and C for apigenin (I), structures A and B for apigenin (II)).

### Vibrational frequency changes upon hydrogen-bond formation

One of the indications of the presence of a hydrogen-bond is the shift of the vibration frequencies and the bond length^25^,^26^. In this work, the frequency analysis was performed using the B3LYP/6−31 + + G(d, p), M062X/6−31 + + G(d, p) and MP2/6−31 + + G(d, p) methods with geometries that were optimized using B3LYP/6−31 + + G(d, p). The representative vibrational frequencies and bond length changes of X−H are shown in Tables 1 and 2. As shown in the tables, the thirty structures of apigenin−H_2_O and apigenin−CH_3_CH_2_OH complexes have one similar characteristic upon hydrogen-bond formation: the X–H bonds are elongated upon complexation, and the elongations of the X–H bonds are concomitant with a decrease in the X–H stretching frequencies (red-shift). For the O−H groups in apigenin, the red-shift and bond elongation are ordered A > B > C for the apigenin (I)−H_2_O/CH_3_CH_2_OH complexes and A > B for the apigenin (II) −H_2_O/CH_3_CH_2_OH complexes. For the O−H groups in H_2_O and CH_3_CH_2_OH, the red-shift and bond elongation are D > E > F > G for apigenin (I) −H_2_O/CH_3_CH_2_OH complexs and C > D > E > F > G for apigenin (II) −H_2_O/CH_3_CH_2_OH complexs. It is clear that the more red-shift and bond elongation values are related to the more stable apigenin−H_2_O/CH_3_CH_2_OH complex and the hydrogen-bonds in the apigenin–H_2_O/CH_3_CH_2_OH complexes are proper red-shift hydrogen-bonds (the vibrational frequency of X−H bond moves to lower wavenumber upon X−H···Y hydrogen-bond formation).

### NBO analysis of apigenin−H_2_O/CH_3_CH_2_OH complexes

Electron density rearrangement accompanies the formation of a hydrogen bond. NBO analysis provides an efficient method for studying intermolecular interactions and a convenient basis for investigating charge transfer in molecular systems. Herein, based on the optimized geometries using the B3 LYP/6−31 + + G(d, p) method, charge distributions of the apigenin−H_2_O/CH_3_CH_2_OH hydrogen-bonded complexes were examined in terms of the NBO analysis using the B3 LYP/6−31 + + G(d, p), M062X/6−31 + + G(d, p) and MP2/6−31 + + G(d, p) methods.

In most of the hydrogen-bond complexes, charge donating centers and charge accepting centers are often the lone pairs [in this work, the lone pairs belong to an oxygen atom, represented as LP(O)] of the hydrogen-bond acceptor and the anti-bonding orbitals of the hydrogen-bond donor (σ*X−H), respectively^27^. In the apigenin−H_2_O/CH_3_CH_2_OH complexes, the electron density change of the σ*X−H and the corresponding interacting stabilization energies computed using the second-order perturbation theory [E(2)] are listed in Tables 1, 2 and 3. As shown in the tables, the electron density and the E(2) in the related localized anti-bonding orbital are increased upon hydrogen-bond formation. These results suggest strong orbital interactions between the LP(O) and the σ*X−H. The increase in the electron density of σ*X−H is a result of the charge transfer from LP(O). This in turn results in the elongation of the X–H bond and a red-shift of the *v*(X−H) bond upon the apigenin−H_2_O/CH_3_CH_2_OH hydrogen-bonded complex formation as shown in Tables 1 and 2. Also shown in the tables, the electron densities change and the E(2) for the X−H···O bonds of structures A, B and C in apigenin (I)−H_2_O/CH_3_CH_2_OH and structure A and B in apigenin (II)−H_2_O/CH_3_CH_2_OH were much larger than the other conformers.

The sum of the atomic charges of each monomer in the complex systems could be defined as a charge transfer (CT) value. Here we observe a charge change in apigenin as the selected monomer for acquiring the CT amounts. Listed in Table 4 are the calculated charge changes of apigenin from complexes to a monomer. The negative value of the charge change means apigenin obtains more charge upon hydrogen-bond formation. The larger absolute value of the charge change indicates a larger CT. As shown in the table, apigenin gets a charge when it acts as a hydrogen-bond donor (structures A, B and C for apigenin (I), structures A, B for apigenin (II)). However, when it is hydrogen-bond acceptor, it loses its charge (structures E and H for apigenin (I), structures F, G for apigenin (II)). Electron transfers occur from the hydrogen-bond acceptor to the hydrogen-bond donor. The stronger hydrogen-bond causes the hydrogen-bond donor to lose more charge. Additionally, as shown in Table 4, when apigenin acts as an electron donor or acceptor only, the stronger interactions will make the charge transfer larger (absolute value: A > B > C > E > H for apigenin (I), A > B > F > G for apigenin (II)).

### AIM analysis of apigenin−H_2_O/CH_3_CH_2_OH complexes

The topological analysis of the electron density further validates the existence of hydrogen-bonds in all of the structures. The following parameters were analyzed: the electron density (*ρ*), the Laplacian of the electron density (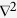
*ρ*), and the energy density (*H*) at the bond critical points (BCPs). All of the parameters were evaluated using the AIM (atoms in molecules) approach with the B3 LYP/6−31 + + G(d, p), M062X/6−31 +  + G(d, p) and MP2/6−31 + + G(d, p) methods. The results are shown in Tables 5 and 6.

It can be observed that the values of *ρ*_BCPs_ are in the range of 0.0063−0.0314 au and 0.0059−0.0354 au for apigenin−H_2_O and apigenin−CH_3_CH_2_OH complexes, respectively, which are within the range of 0.002−0.04 au that was determined for hydrogen-bonds^28^. The values of 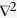
*ρ*_BCPs_ are all positive, ranging from 0.0346 to 0.1047 au for apigenin−H_2_O complexes and 0.0304 to 0.1161 au for apigenin−CH_3_CH_2_OH complexes. These values are within the range of 0.020−0.139 au that was determined for hydrogen-bond-including complexes^28^, thus indicating the formation of hydrogen-bond interactions in these complexes. It is well known that the higher value of *ρ*_BCPs_ and the sum of 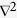
*ρ*_BCPs_ implies stronger interactions. Therefore, as the results of Tables 5 and 6 shown, the *ρ*_BCPs_ of A is the greatest and is in agreement with its highest interaction energy, followed by B, C, D, E, F, G and H.

As previously noted, *H*_BCPs_ is a more appropriate index used to gain a deeper understanding of the non-covalent interactions^29^. When *H*_BCPs_ < 0, the hydrogen-bond possesses an interaction of a dominantly covalent character. When *H*_BCPs_ > 0, the hydrogen-bond is electrostatically dominant. As shown in Tables 5 and 6, in apigenin (I)−H_2_O/CH_3_CH_2_OH complexes, it is evident that for the hydrogen-bonds in conformers A, B, C, E and G, C8−H6···O in D, and C12−H8···O, they are essentially electrostatic in nature. In apigenin (II)−H_2_O/CH_3_CH_2_OH complexes, for the hydrogen-bonds in conformers A, B, D and E and C8−H6···O in C, they are essentially electrostatic in nature. However, negative values of *H*_BCPs_ are obtained for C9−O4···H, C13−O2···H and C4−O5···H in apigenin−H_2_O/CH_3_CH_2_OH complexes, suggesting that the corresponding hydrogen-bonds in these conformers have some degree of covalent character.

## Conclusions

In this work, the hydrogen-bonding interactions between ethanol/water and apigenin were investigated from a theoretical perspective using quantum chemical calculations. Two conformations of the apigenin molecule were considered in this work. The equilibrium structures were analyzed using the B3LYP/6−31 + + G(d, p) method. Based on the optimized geometries from the B3LYP/6−31 + + G(d, p) method, the molecular energies, charges, vibrational frequencies, NBO analysis and topological analysis were analyzed using the B3LYP/6−31 + + G(d, p), the M062X/6−31 + + G(d, p) and the MP2/6−31 + + G(d, p) methods. The main conclusions are as follows:

1. For the apigenin monomer: the chromone part is in the plane, whereas the phenyl substituent is out of the plane with the chromone part. Therefore, the molecular structure of apigenin is non-planar. All of the hydrogen atoms can be used as hydrogen-bond donors, and the oxygen atoms can act as hydrogen-bond acceptors.
2. Eight and seven optimized geometries were obtained for apigenin (I)−H_2_O/CH_3_CH_2_OH and apigenin (II)−H_2_O/CH_3_CH_2_OH complexes, respectively. All the oxygen atoms can be used as hydrogen-bond acceptors, and all the hydrogen atoms in the chromone part can be hydrogen-bond donors. However, the aromatic hydrogen atoms in the phenyl substituent did not form hydrogen-bonds with H_2_O and CH_3_CH_2_OH. H_2_O and CH_3_CH_2_OH formed strong hydrogen-bonds with the hydrogen atoms of the hydroxyl groups and the oxygen atoms of the carbonyl group of apigenin.
3. The formation of apigenin−H_2_O/CH_3_CH_2_OH complexes is accompanied by charge rearrangement. The electron density and the E(2) in the related localized anti-bonding orbital are increased upon hydrogen-bond formation. These are the cause of the elongation and red-shift of the X−H bond. In the apigenin−H_2_O/CH_3_CH_2_OH complexes, the sum of charge change transfers from the hydrogen-bond acceptor to the hydrogen-bond donor. The stronger interaction makes the charge change larger than observed in the less stable complexes.
4. According to the topology analysis performed using the AIM theory, the C9−O4···H, C13−O2···H and C4−O5···H hydrogen-bonds have some degree of covalent character. However, the other hydrogen-bonds are electrostatic in nature. Furthermore, the hydroxyl groups are the preferred hydrogen-bonding sites.

## Methods

### Computational details

All of the calculations were performed using the Gaussian 09 program^30^. The geometries of the monomers and apigenin−H_2_O/CH_3_CH_2_OH complexes were optimized by B3LYP/6–31 + + G(d, p). The most stable optimized geometries at a local energy minimum were verified by the lack of any imaginary vibrational frequency. The molecular energies, charge, and vibrational frequencies of the monomers and apigenin–H_2_O/CH_3_CH_2_OH complexes were calculated using the B3LYP/M062X/MP2 methods with the 6–31 + + G(d, p) basis set using the optimized geometries of the B3LYP/6−31 + + G(d, p) method. The interaction energy in the complexes can be regarded as the energetic difference between the complex and the sum of the individual monomers:


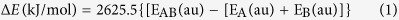


In Equation (1), 2625.5 is unit conversion from au to kJ/mol. Moreover, the calculation of the interaction energy is corrected by the basis set superposition error (BSSE) correction according to the counterpoise procedure of Boys and Bernardi^31^.

To better understand the nature of the intermolecular hydrogen-bonding interactions in the apigenin−H_2_O/CH_3_CH_2_OH complexes, NBO^27^ and AIM^32^ analyses were also carried out at the B3LYP/6−31 + + G(d, p), M062X/6−31 + + G(d, p) and MP2/6−31 + + G(d, p) levels. NBO analysis was performed to elucidate charge transfer upon hydrogen-bond formation. The NBO program as implemented in the Gaussian 09 package is used to do the NBO analysis. In the NBO analysis, the donor and acceptor interactions could be estimated through the second-order perturbation theory, described using the following equation:


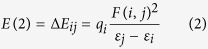


where *q*_i_ is the donor orbital occupancy, *ε*_j_ and *ε*_i_ are diagonal elements, and *F*(i, j) is the off-diagonal NBO Fock matrix element.

In the AIM analysis, the search of BCPs and a detailed topological analysis was performed with the Multiwfn 3.3.8 suite^33^. The topological parameters, such as *ρ*, 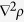 and *H* at the BCPs, were used to predict the nature of the hydrogen-bonding interaction.

## Additional Information

**How to cite this article**: Zheng, Y.-Z. *et al*. Hydrogen-bonding Interactions between Apigenin and Ethanol/Water: A Theoretical Study. *Sci. Rep*. **6**, 34647; doi: 10.1038/srep34647 (2016).

## Supplementary Material

Supplementary Information

## Figures and Tables

**Table 1:** The wavenumber, bond length and electron density (in σ*X−H) changes of the X−H bonds in apigenin−H_2_O complexes from different complexes to monomers at the B3LYP/6−31 + + G(d, p), M062X/6−31 + + G(d, p) and MP2/6−31 + + G(d, p) levels.

	bond	wavenumber				bond length		electron density	
		B3LYP	M062X	MP2	B3LYP	M062X	MP2	B3LYP	M062X
Apigenin (I)−H_2_O									
**A**	O2−H9	−234	−255	−264	0.012	0.014	0.014	0.031	0.027
**B**	O5−H3	−226	−245	−255	0.011	0.012	0.013	0.030	0.026
**C**	O3−H7	−224	−233	−245	0.011	0.012	0.011	0.031	0.027
**D**	O−H	−41	−39	−38	0.008	0.007	0.007	0.019	0.016
	C8−H6	−0.6	−0.7	−0.8	9E−05	9E−05	9E−05	0.003	0.003
**E**	O−H	−39	−38	−36	0.004	0.003	0.003	0.009	0.007
**F**	O−H	−27	−25	−23	0.002	0.002	0.002	0.006	0.005
	C12−H8	−2	−2	−2	3E−04	4E−04	4E−04	0.004	0.003
**G**	O−H	−25	−22	−20	0.002	0.002	0.002	0.004	0.004
	C14−H10	−3	−3	−3	1E−05	1E−05	1E−05	0.003	0.003
**H**	O−H	−29	−27	−25	0.002	0.002	0.002	0.006	0.005
Apigenin (II)−H_2_O									
A	O5−H3	−234	−245	−250	0.012	0.014	0.014	0.030	0.026
B	O2−H9	−231	−240	−248	0.012	0.014	0.014	0.030	0.026
C	C8−H6	−2	−3	−4	3E−04	3E−04	4E−04	0.003	0.003
C	O−H	−39	−37	−36	0.007	0.007	0.007	0.030	0.025
D	C12−H8	−10	−12	−13	0.001	0.001	0.002	0.002	0.001
D	O−H	−34	−33	−31	0.003	0.003	0.002	0.017	0.014
E	C14−H10	−0.3	−0.4	−0.5	2E−04	2E−04	2E−04	0.003	0.002
E	O−H	−27	−26	−24	0.002	0.002	0.002	0.015	0.012
F	O−H	−25	−25	−23	0.002	0.002	0.002	0.012	0.010
G	O−H	−22	−21	−20	0.001	0.001	0.001	0.011	0.006

The non-numbered hydrogen and oxygen atoms are the corresponding atoms in H_2_O.

The wavenumber change of O−H in H_2_O is an asymmetrical stretching vibration.

**Table 2:** The wavenumber, bond length and electron density (in σ*X−H) changes of the X−H bonds in apigenin−CH_3_CH_2_OH complexes from different complexes to monomers at the B3LYP/6−31 + + G(d, p), M062X/6−31 + + G(d, p) and MP2/6−31 + + G(d, p) levels.

	bond	wavenumber				bond length		electron density	
		B3LYP	M062X	MP2	B3LYP	M062X	MP2	B3LYP	M062X
Apigenin (I)−CH_3_CH_2_OH									
**A**	O2−H9	−319	−333	−336	0.016	0.017	0.018	0.039	0.035
**B**	O5−H3	−310	−328	−330	0.015	0.016	0.017	0.038	0.034
**C**	O3−H7	−308	−318	−325	0.015	0.016	0.016	0.039	0.035
**D**	O−H	−303	−313	−316	0.014	0.015	0.015	0.039	0.033
	C8−H6	−5	−5	−5	3E−04	4E−04	4E−04	0.003	0.003
**E**	O−H	−146	−158	−160	0.009	0.010	0.011	0.019	0.016
**F**	O−H	−63	−74	−77	0.004	0.005	0.006	0.011	0.010
	C12−H8	−5	−6	−6	5E−04	6E−04	5E−04	0.004	0.004
**G**	O−H	−41	−46	−48	0.003	0.003	0.003	0.007	0.007
	C14−H10	−6	−6	−6	6E−04	7E−04	6E−04	0.006	0.005
**H**	O−H	−66	−68	−69	0.004	0.004	0.004	0.013	0.011
Apigenin (II)−CH_3_CH_2_OH									
**A**	O5−H3	−394	−399	−412	0.018	0.019	0.020	0.040	0.035
**B**	O2−H9	−384	−390	−401	0.018	0.018	0.019	0.039	0.034
**C**	C8−H6	−15	−20	−26	5E−04	6E−04	7E−04	0.004	0.004
**C**	O−H	−242	−255	−269	0.013	0.014	0.015	0.032	0.027
**D**	C12−H8	−23	−29	−33	0.002	0.003	0.003	0.003	0.002
**D**	O−H	−96	−106	−110	0.006	0.007	0.008	0.017	0.014
**E**	C14−H10	−24	−30	−33	0.001	0.001	0.001	0.005	0.004
**E**	O−H	−60	−74	−81	0.004	0.005	0.006	0.007	0.006
**F**	O−H	−55	−65	−73	0.003	0.004	0.005	0.012	0.012
**G**	O−H	−37	−42	−44	0.002	0.002	0.002	0.011	0.011

The non-numbered hydrogen and oxygen atoms are the corresponding atoms in CH_3_CH_2_OH.

**Table 3:** Second-order perturbation theory analysis of the Fock matrix in the NBO basis for apigenin−H_2_O/CH_3_CH_2_OH.

	donor (i)	acceptor (j)	apigenin−H_2_O		apigenin−CH_3_CH_2_OH	
			B3LYP	M062X	B3LYP	M062X
Apigenin (I)−H_2_O						
**A**	LP(1)O	σ*(1)O2−H9	24.69	27.55	5.04	5.19
	LP(2)O	σ*(1)O2−H9	15.99	20.44	28.73	30.15
**B**	LP(1)O	σ*(1)O5−H3	10.24	11.21	1.69	1.77
	LP(2)O	σ*(1)O5−H3	17.26	18.19	18.61	19.77
**C**	LP(1)O	σ*(1)O3−H7	8.09	11.11	0.77	0.96
	LP(2)O	σ*(1)O3−H7	18.38	19.36	18.82	20.12
**D**	LP (1)O4	σ*(1)O−H	18.63	28.87	10.99	11.58
	LP(2)O4	σ*(1)O−H	1.38	1.25	0.45	0.53
	LP(2)O	σ*(1)C8−H6	1.41	1.54	2.25	2.00
**E**	LP(1)O4	σ*(1)O −H	3.45	3.72	12.45	16.71
	LP(2)O4	σ*(1)O−H	14.94	16.78	3.52	5.67
	LP(1)O3	σ*(1)O1−H	1.22	1.34	1.23	1.67
**F**	LP(1)O	σ*(1)C12−H8	12.92	11.65	1.06	1.17
	LP(2)O	σ*(1)C12−H8	3.03	3.13	10.55	10.57
	LP(1)O2	σ*(1)O−H	4.73	4.92	0.86	0.92
**G**	LP(1)O	σ*(1)C14−H10	13.74	14.01	1.57	1.63
	LP(2)O	σ*(1)C14−H10	4.13	3.94	11.01	11.12
	LP(1)O1	σ*(1)O−H	1.73	1.91	0.24	0.25
	LP(2)O1	σ*(1)O−H	0.29	0.35	2.07	2.35
**H**	LP (1)O5	σ*(1)O−H	5.48	5.74	2.16	2.17
Apigenin (II)−CH_3_CH_2_OH						
**A**	LP(1)O	σ*(1)O5−H3	30.14	30.12	1.65	1.72
	LP(2)O	σ*(1)O5−H3	17.81	18.77	19.27	20.52
**B**	LP(1)O	σ*(1)O2−H9	24.22	25.13	3.13	5.49
	LP(2)O	σ*(1)O2−H9	11.65	13.35	12.03	18.94
**C**	LP (1)O4	σ*(1)O−H	0.69	2.21	7.31	7.96
	LP(2)O4	σ*(1)O−H	13.14	11.76	16.75	16.64
	LP(1)O	σ*(1)C8−H6	0.18	0.22	1.39	1.99
	LP(2)O	σ*(1)C8−H6	1.8	1.94	1.27	1.34
**D**	LP (1)O4	σ*(1)O−H	7.78	8.35	2.62	2.97
	LP(2)O4	σ*(1)O−H	0.11	0.23	0.06	0.11
	LP(1)O	σ*(1)C12−H8	1.23	1.61	0.18	0.20
	LP(2)O	σ*(1)C12−H8	0.21	0.44	0.55	0.59
**E**	LP (1)O1	σ*(1)O−H	6.81	6.92	5.81	5.91
	LP(2)O1	σ*(1)O−H	0.03	0.05	0.16	0.22
	LP(1)O	σ*(1)C14−H10	0.23	0.27	0.63	0.67
	LP(2)O	σ*(1)C14−H10	1.76	1.84	1.34	1.45
**F**	LP(1)O5	σ*(1)O−H	3.12	3.27	2.93	3.15
	LP(2)O5	σ*(1)O−H	0.11	0.14	0.12	0.13
**G**	LP(1)O2	σ*(1)O−H	2.18	2.44	2.55	2.79
	LP(2)O2	σ*(1)O−H	0.06	0.07	0.11	0.20

The non-numbered hydrogen and oxygen atoms are the corresponding atoms in H_2_O and CH_3_CH_2_OH.

**Table 4:** Charge (q in e) changes of apigenin from different complexes to monomers obtained using the NBO theory at the B3LYP/6−31 + + G(d, p), M062X/6−31 + + G(d, p) and MP2/6−31 + + G(d, p) levels.

	apigenin–H_2_O			apigenin–CH_3_CH_2_OH		
	B3LYP	M062X	MP2	B3LYP	M062X	MP2
Apigenin (I)−H_2_O						
A	−0.034	−0.028	−0.031	−0.042	−0.037	−0.041
B	−0.032	−0.027	−0.030	−0.041	−0.036	−0.035
C	−0.031	−0.026	−0.029	−0.039	−0.035	−0.034
D	0.030	0.026	0.028	0.034	0.029	0.030
E	0.015	0.013	0.014	0.019	0.016	0.018
F	0.005	0.004	0.004	0.005	0.004	0.004
G	−1E−04	−2E−04	−6E−04	−0.004	−0.003	−0.004
H	0.008	0.006	0.007	0.008	0.007	0.007
Apigenin (II)−CH_3_CH_2_OH						
A	−0.031	−0.027	−0.158	−0.041	−0.036	−0.137
B	−0.031	−0.027	−0.157	−0.041	−0.036	−0.041
C	0.025	0.020	0.023	0.025	0.021	0.023
D	0.013	0.011	0.012	0.012	0.011	0.011
E	−0.001	−0.001	−0.065	−0.004	−0.003	−0.004
F	0.006	0.005	0.006	0.006	0.005	0.006
G	0.012	0.009	0.011	0.012	0.009	0.010

**Table 5:** The electron density (*ρ*), the Laplacian of the electron density (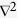
*ρ*), and the energy density (*H*) at the bond critical points (BCPs) of the hydrogen-bonds of apigenin−H_2_O complexes, obtained using the AIM theory at the B3LYP/6−31 + + G(d, p), M062X/6−31 + + G(d, p) and MP2/6−31 + + G(d, p) levels.

	hydrogen bonds	*ρ*_BCPs_(10^−1^)			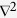 *ρ*_BCPs_(10^−1^)			*H*_BCPs_(10^−3^)		
		B3LYP	M062X	MP2	B3LYP	M062X	MP2	B3LYP	M062X	MP2
Apigenin (I)−H_2_O										
**A**	O2−H9···O	0.313	0.301	0.285	0.945	1.010	1.043	0.408	0.943	1.080
**B**	O5−H3···O	0.308	0.297	0.281	0.928	0.993	1.025	0.364	0.886	1.002
**C**	O3−H7···O	0.306	0.295	0.280	0.934	0.999	1.021	0.169	0.693	0.868
**D**	C9−O4···H	0.304	0.290	0.276	0.945	1.021	1.065	−0.423	−0.262	−0.273
**D**	C8−H6···O	0.099	0.100	0.096	0.378	0.386	0.395	1.176	1.120	1.167
**E**	C11−O3···H	0.127	0.128	0.123	0.520	0.526	0.538	1.063	0.943	1.023
**E**	C9−O4···H	0.205	0.199	0.187	0.630	0.658	0.664	0.006	0.125	0.500
**F**	C13−O2···H	0.190	0.185	0.177	0.583	0.607	0.612	−0.171	−0.134	−0.089
**F**	C12−H8···O	0.101	0.101	0.097	0.349	0.357	0.367	0.800	0.760	0.831
**G**	C14−H10···O	0.099	0.099	0.096	0.356	0.363	0.372	0.902	0.856	0.904
**G**	C7−O1···H	0.132	0.131	0.124	0.422	0.431	0.440	0.098	0.009	0.106
**H**	C4−O5···H	0.202	0.197	0.188	0.615	0.643	0.648	−0.187	−0.107	−0.196
Apigenin (II)−H_2_O										
**A**	O5−H3···O	0.314	0.302	0.286	0.947	1.012	1.047	0.407	0.943	2.092
**B**	O2−H9···O	0.312	0.301	0.284	0.936	1.001	1.034	0.324	0.855	1.993
**C**	C9−O4···H	0.304	0.293	0.280	0.858	0.926	0.957	−0.564	−0.087	−0.772
**C**	C8−H6···O	0.103	0.104	0.100	0.384	0.392	0.402	1.091	1.032	1.078
**D**	C11−O3···H	0.232	0.234	0.214	0.697	0.715	0.746	0.190	0.170	0.174
**D**	C12−H8···O	0.067	0.069	0.063	0.246	0.262	0.260	0.978	0.898	1.042
**E**	C14−H10···O	0.200	0.199	0.196	0.346	0.353	0.363	0.814	0.778	0.838
**E**	C7−O1···H	0.121	0.120	0.114	0.401	0.409	0.418	0.273	0.191	0.267
**F**	C4−O5···H	0.195	0.190	0.182	0.597	0.623	0.627	−0.177	−0.126	−0.120
**G**	C13−O2···H	0.216	0.216	0.198	0.650	0.650	0.688	−0.136	−0.136	−0.193

The non-numbered hydrogen and oxygen atoms are the corresponding atoms in H_2_O.

**Table 6:** The electron density (*ρ*), the Laplacian of electron density (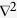
*ρ*), and the energy density (*H*) at the bond critical points (BCPs) of the hydrogen-bonds of apigenin−CH_3_CH_2_OH complexes, obtained using the AIM theory at the B3LYP/6−31 + + G(d, p), M062X/6−31 + + G(d, p) and MP2/6−31 + + G(d, p) levels.

	hydrogen bonds	*ρ*_BCPs_(10^−1^)			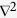 *ρ*_BCPs_(10^−1^)			*H*_BCPs_(10^−3^)		
		B3LYP	M062X	MP2	B3LYP	M062X	MP2	B3LYP	M062X	MP2
Apigenin (I)−CH_3_CH_2_OH										
**A**	O2−H9···O	0.352	0.339	0.321	1.031	1.104	1.155	0.059	0.697	0.997
**B**	O5−H3···O	0.347	0.334	0.316	1.012	1.086	1.135	0.016	0.651	0.932
**C**	O3−H7···O	0.346	0.333	0.315	1.028	1.101	1.125	0.134	0.490	0.846
**D**	C9−O4···H	0.331	0.318	0.302	0.930	1.004	1.049	−0.498	−0.276	−0.181
**D**	C8−H6···O	0.092	0.929	0.090	0.356	0.363	0.373	1.193	1.147	1.129
**E**	C11−O3···H	0.121	0.121	0.116	0.502	0.507	0.522	1.130	1.009	1.101
**E**	C9−O4···H	0.212	0.205	0.193	0.647	0.677	0.686	0.013	0.165	0.591
**F**	C13−O2···H	0.183	0.179	0.179	0.557	0.578	0.578	−0.218	−0.197	−0.197
**F**	C12−H8···O	0.095	0.095	0.095	0.336	0.343	0.343	0.841	0.804	0.804
**G**	C14−H10···O	0.110	0.109	0.105	0.351	0.359	0.372	0.552	0.523	0.582
**G**	C7−O1···H	0.082	0.815	0.077	0.306	0.311	0.321	0.788	0.735	0.820
**H**	C4−O5···H	0.196	0.190	0.181	0.587	0.611	0.617	−0.271	−0.208	−0.084
Apigenin (II)−CH_3_CH_2_OH										
**A**	O5−H3···O	0.354	0.341	0.323	1.034	1.109	1.161	0.032	0.671	1.999
**B**	O2−H9···O	0.350	0.338	0.320	1.021	1.096	1.146	0.007	0.643	1.941
**C**	C9−O4···H	0.293	0.282	0.269	0.814	0.877	0.907	−0.729	−0.290	−0.506
**C**	C8−H6···O	0.102	0.102	0.099	0.371	0.379	0.390	1.011	0.990	0.956
**D**	C11−O3···H	0.224	0.217	0.206	0.665	0.701	0.710	0.305	0.139	0.295
**D**	C12−H8···O	0.062	0.062	0.059	0.224	0.230	0.240	0.948	0.974	1.030
**E**	C14−H10···O	0.205	0.204	0.200	0.336	0.344	0.357	0.579	0.561	0.626
**E**	C7−O1···H	0.080	0.080	0.075	0.304	0.309	0.319	0.802	0.755	0.831
**F**	C4−O5···H	0.189	0.184	0.176	0.572	0.594	0.599	−0.248	−0.208	−0.392
**G**	C13−O2···H	0.188	0.183	0.173	0.564	0.591	0.585	−0.269	−0.146	−0.219

The non-numbered hydrogen and oxygen atoms are the corresponding atoms in CH_3_CH_2_OH.
