# Supplementary material for: Hydrogen-bonding Interactions between Apigenin and Ethanol/Water: A Theoretical Study
Source: Sci Rep. 2016 Oct 4;6:34647. doi: 10.1038/srep34647 (PMC5048163; doi:10.1038/srep34647)
Supplement: Supplementary Information [file srep34647-s1.doc]

**Supplementary Information for:**

**Hydrogen-bonding Interactions between Apigenin and Ethanol/Water: A Theoretical Study**

Yan-Zhen Zheng,^a*^ Yu Zhou,^b^ Qin Liang,^a^ Da-Fu Chen^a*^, Rui Guo^a^ & Rong-Cai Lai^a^

*^a^**College of Bee Science,* *Fujian Agriculture and Forestry University, Fuzhou 350002, P. R. China*

*^b^**Key Laboratory of Bioorganic Phosphorous Chemistry and Chemical Biology (Ministry of Education), Department of Chemistry, Tsinghua University, Beijing 100084, P. R. China*

***To whom correspondence should be addressed.**

Dr. Yan-Zhen Zheng, Da-Fu Chen

Tel. (+86) 591 83789482

Fax. (+86) 591 83789482

Email: [zhengyz15@gmail.com](mailto:yuzhw@tsinghua.edu.cn)

dfchen826@163.com

**Table S1.** Optimized parameter values of the apigenin (I) monomer [bond length in (Å), bond and dihedral angles in (◦)] obtained by the B3LYP/6−31++G(d, p), M062X/6−31++G(d, p) and MP2/6−31++G(d, p) methods.

| Bond length | B3LYP | M062X | MP2 | Bond angle | B3LYP | M062X | MP2 | Dihedral angle | B3LYP | M062X | MP2 |
| --- | --- | --- | --- | --- | --- | --- | --- | --- | --- | --- | --- |
| C1−C2 | 1.408 | 1.402 | 1.406 | C1−C2−C3 | 121.2 | 120.9 | 120.5 | C1−C2−C3−C4 | -0.250 | -0.385 | -0.298 |
| C2−C3 | 1.390 | 1.387 | 1.392 | C1−C2−H1 | 119.4 | 119.4 | 119.6 | C2−C3−C4−C5 | -0.263 | -0.220 | -0.008 |
| C3−C4 | 1.399 | 1.396 | 1.399 | H1−C2−C3 | 119.3 | 119.7 | 119.9 | C3−C4−C5−C6 | 0.198 | 0.237 | 0.473 |
| C4−C5 | 1.401 | 1.396 | 1.399 | C2−C3−C4 | 119.8 | 119.7 | 119.8 | C3−C2−C1−C7 | -178.9 | -178.7 | -179.2 |
| C5−C6 | 1.392 | 1.389 | 1.395 | C2−C3−H2 | 121.1 | 121.4 | 121.2 | C6−C1−C2−H1 | -179.7 | -179.6 | -179.7 |
| C1−C6 | 1.405 | 1.398 | 1.403 | H2−C3−C4 | 119.1 | 118.9 | 118.9 | C1−C2−C3−H2 | 179.5 | 179.4 | 179.5 |
| C1−C7 | 1.472 | 1.473 | 1.467 | C3−C4−C5 | 119.8 | 120.0 | 120.2 | C2−C3−C4−O5 | -180.0 | -179.9 | -179.9 |
| C2−H1 | 1.084 | 1.085 | 1.083 | C3−C4−O5 | 117.4 | 117.3 | 116.9 | C3−C4−C5−H4 | -179.0 | -178.9 | -179.7 |
| C3−H2 | 1.085 | 1.084 | 1.082 | O5−C4−C5 | 122.7 | 122.7 | 122.9 | C3−C4−O5−H3 | -179.8 | 180.0 | -179.7 |
| C4−O5 | 1.366 | 1.359 | 1.375 | C4−O5−H3 | 110.3 | 110.4 | 109.4 | C4−C5−C6−H5 | -178.6 | -178.4 | -178.9 |
| O5−H3 | 0.966 | 0.964 | 0.967 | C4−C5−C6 | 119.9 | 119.8 | 119.8 | C2−C1−C7−C8 | 160.1 | 156.2 | 153.9 |
| C5−H4 | 1.088 | 1.087 | 1.085 | C4−C5−H4 | 120.2 | 120.2 | 120.2 | C1−C7−C8−C9 | 179.5 | 179.6 | 179.1 |
| C6−H5 | 1.085 | 1.085 | 1.083 | H4−C5−C6 | 119.9 | 120.0 | 119.9 | C7−C8−C9−C10 | 1.428 | 1.288 | 1.621 |
| C7−C8 | 1.354 | 1.347 | 1.356 | C5−C6−C1 | 121.1 | 120.8 | 120.5 | C8−C9−C10−C15 | -0.600 | -0.495 | -0.566 |
| C8−C9 | 1.463 | 1.465 | 1.458 | C5−C6−H5 | 118.8 | 119.2 | 119.6 | C2−C1−C7−O1 | -19.23 | -23.09 | -25.14 |
| C9−C10 | 1.482 | 1.481 | 1.481 | H5−C6−C1 | 120.1 | 120.0 | 119.9 | C1−C7−C8−H6 | -1.542 | -1.800 | -1.513 |
| C10−C15 | 1.408 | 1.401 | 1.406 | C6−C1−C2 | 118.1 | 118.7 | 119.1 | C7−C8−C9−O4 | -179.0 | -179.3 | -179.3 |
| C15−O1 | 1.370 | 1.363 | 1.379 | C6−C1−C7 | 120.9 | 120.7 | 120.3 | C8−C9−C10−C11 | 179.2 | 179.3 | 179.2 |
| C7−O1 | 1.369 | 1.360 | 1.370 | C7−C1−C2 | 121.0 | 120.6 | 120.5 | C9−C10−C15−C14 | 179.8 | 179.8 | 179.7 |
| C8−H6 | 1.082 | 1.083 | 1.081 | C1−C7−C8 | 126.8 | 126.1 | 125.8 | C9−C10−C11−O3 | 0.177 | 0.217 | 0.125 |
| C9−O4 | 1.232 | 1.221 | 1.242 | C7−C8−H6 | 120.6 | 120.6 | 119.8 | C10−C11−C12−H8 | -180.0 | -179.9 | -179.5 |
| C10−C11 | 1.423 | 1.418 | 1.420 | C7−C8−C9 | 123.1 | 122.5 | 122.4 | C11−C12−C13−O2 | -179.9 | -179.8 | -179.7 |
| C11−C12 | 1.393 | 1.389 | 1.391 | H6−C8−C9 | 116.3 | 116.9 | 117.7 | C10−C15−C14−H10 | 179.7 | 179.8 | 179.7 |
| C12−C13 | 1.400 | 1.397 | 1.400 | C8−C9−C10 | 114.1 | 113.8 | 113.8 | C12−C13−O2−H9 | 179.8 | 178.8 | 179.6 |
| C13−C14 | 1.392 | 1.387 | 1.388 | C8−C9−O4 | 121.3 | 121.5 | 121.7 | C10−C11−O3−H7 | -179.9 | -179.9 | -179.5 |
| C14−C15 | 1.397 | 1.394 | 1.398 | O4−C9−C10 | 124.6 | 124.6 | 124.5 | O1−C7−C8−C9 | -1.217 | -1.176 | -1.014 |
| C11−O3 | 1.354 | 1.347 | 1.365 | C9−C10−C15 | 119.1 | 119.3 | 119.1 | C9−C10−C15−O1 | -0.423 | -0.423 | -0.420 |
| O3−H7 | 0.987 | 0.985 | 0.969 | C9−C10−C11 | 124.6 | 124.1 | 124.3 | C10−C15−O1−C7 | 0.740 | 0.642 | 0.838 |
| C12−H8 | 1.086 | 1.085 | 1.084 | C11−C10−C15 | 116.3 | 116.6 | 116.2 | C11−C12−C13−C14 | 0.060 | 0.123 | 0.091 |
| C13−O2 | 1.364 | 1.356 | 1.373 | C10−C15−O1 | 122.0 | 122.1 | 122.5 | C13−C14−C15−C10 | 0.014 | 0.028 | 0.022 |
| O2−H9 | 0.966 | 0.964 | 0.967 | C10−C15−C14 | 123.6 | 123.5 | 123.6 | C12−C13−C14−C15 | -0.041 | -0.093 | -0.091 |
| C14−H10 | 1.085 | 1.085 | 1.089 | C14−C15−O1 | 114.4 | 114.4 | 113.8 | C13−C14−C15−O1 | -179.7 | -179.7 | -179.7 |
|  |  |  |  | C15−O1−C7 | 120.8 | 120.4 | 119.0 | C14−C15−C10−C11 | -0.005 | 0.006 | -0.004 |
|  |  |  |  | C10−C11−C12 | 121.1 | 121.0 | 121.0 | C12−C13−C14−H10 | -179.7 | -179.9 | -179.8 |
|  |  |  |  | C10−C11−O3 | 118.2 | 117.9 | 117.8 |  |  |  |  |
|  |  |  |  | O3−C11−C12 | 120.7 | 121.0 | 121.1 |  |  |  |  |
|  |  |  |  | C11−O3−H7 | 109.4 | 109.6 | 108.3 |  |  |  |  |
|  |  |  |  | C11−C12−C13 | 120.1 | 120.0 | 120.1 |  |  |  |  |
|  |  |  |  | C11−C12−H8 | 120.9 | 121.2 | 120.9 |  |  |  |  |
|  |  |  |  | H8−C12−C13 | 119.0 | 118.9 | 118.8 |  |  |  |  |
|  |  |  |  | C12−C13−C14 | 120.8 | 121.0 | 120.8 |  |  |  |  |
|  |  |  |  | C12−C13−O2 | 116.6 | 116.5 | 116.2 |  |  |  |  |
|  |  |  |  | O2−C13−C14 | 122.5 | 122.5 | 122.9 |  |  |  |  |
|  |  |  |  | C13−O2−H9 | 110.2 | 110.3 | 109.3 |  |  |  |  |
|  |  |  |  | C13−C14−C15 | 118.1 | 118.0 | 117.9 |  |  |  |  |
|  |  |  |  | C13−C14−H10 | 122.3 | 122.5 | 122.6 |  |  |  |  |
|  |  |  |  | H10−C14−C15 | 119.6 | 119.5 | 119.4 |  |  |  |  |

**Table S2.** Optimized parameter values of the apigenin (II) monomer [bond length in (Å), bond and dihedral angles in (◦)] obtained by the B3LYP/6−31++G(d, p) , M062X/6−31++G(d, p) and MP2/6−31++G(d, p) methods.

| Bond length | B3LYP | M062X | MP2 | Bond angle | B3LYP | M062X | MP2 | Dihedral angle | B3LYP | M062X | MP2 |
| --- | --- | --- | --- | --- | --- | --- | --- | --- | --- | --- | --- |
| C1−C2 | 1.408 | 1.402 | 1.406 | C1−C2−C3 | 121.2 | 120.9 | 120. 5 | C1−C2−C3−C4 | -0.296 | -0.451 | -0.395 |
| C2−C3 | 1.390 | 1.386 | 1.392 | C1−C2−H1 | 119.5 | 119.4 | 119.6 | C2−C3−C4−C5 | -0.240 | -0.192 | -0.069 |
| C3−C4 | 1.400 | 1.396 | 1.400 | H1−C2−C3 | 119.4 | 119.7 | 119.9 | C3−C4−C5−C6 | 0.232 | 0.297 | 0.374 |
| C4−C5 | 1.401 | 1.397 | 1.399 | C2−C3−C4 | 119.8 | 119.7 | 119.8 | C3−C2−C1−C7 | -178.9 | -178.7 | -179.3 |
| C5−C6 | 1.391 | 1.389 | 1.395 | C2−C3−H2 | 121.1 | 121.4 | 121.2 | C6−C1−C2−H1 | -179.8 | -179.7 | -179.8 |
| C1−C6 | 1.406 | 1.399 | 1.403 | H2−C3−C4 | 119.1 | 118.9 | 119.0 | C1−C2−C3−H2 | 179.5 | 179.3 | 179.5 |
| C1−C7 | 1.471 | 1.472 | 1.466 | C3−C4−C5 | 119.9 | 120.1 | 120.2 | C2−C3−C4−O5 | -180.0 | -179.9 | -179.9 |
| C2−H1 | 1.084 | 1.085 | 1.083 | C3−C4−O5 | 117.4 | 117.2 | 116.8 | C3−C4−C5−H4 | -179.0 | -178.8 | -178.8 |
| C3−H2 | 1.085 | 1.084 | 1.082 | O5−C4−C5 | 122.8 | 122.7 | 122.9 | C3−C4−O5−H3 | -179.8 | 179.8 | -179.9 |
| C4−O5 | 1.365 | 1.357 | 1.374 | C4−O5−H3 | 110.4 | 110.5 | 109.5 | C4−C5−C6−H5 | -178.5 | -178.4 | -178.9 |
| O5−H3 | 0.967 | 0.964 | 0.967 | C4−C5−C6 | 119.9 | 119.8 | 119.8 | C2−C1−C7−C8 | 160.0 | 155.8 | 153.9 |
| C5−H4 | 1.087 | 1.087 | 1.085 | C4−C5−H4 | 120.2 | 120.2 | 120.2 | C1−C7−C8−C9 | 179.7 | 179.8 | 179.8 |
| C6−H5 | 1.085 | 1.085 | 1.083 | H4−C5−C6 | 119.9 | 120.1 | 119.9 | C7−C8−C9−C10 | 1.161 | 1.113 | 1.142 |
| C7−C8 | 1.362 | 1.354 | 1.363 | C5−C6−C1 | 121.0 | 120.8 | 120.4 | C8−C9−C10−C15 | -0.449 | -0.458 | -0.477 |
| C8−C9 | 1.447 | 1.450 | 1.445 | C5−C6−H5 | 118.7 | 119.1 | 119.5 | C2−C1−C7−O1 | -19.32 | -23.47 | -24.85 |
| C9−C10 | 1.454 | 1.456 | 1.457 | H5−C6−C1 | 120.2 | 120.1 | 120.0 | C1−C7−C8−H6 | -1.670 | -1.945 | -1.766 |
| C10−C15 | 1.404 | 1.398 | 1.403 | C6−C1−C2 | 118.2 | 118.8 | 119.2 | C7−C8−C9−O4 | -179.4 | -179.6 | -176.9 |
| C15−O1 | 1.373 | 1.365 | 1.380 | C6−C1−C7 | 120.9 | 120.7 | 120.3 | C8−C9−C10−C11 | 179.2 | 179.2 | 179.3 |
| C7−O1 | 1.365 | 1.356 | 1.366 | C7−C1−C2 | 120.9 | 120.6 | 120.5 | C9−C10−C15−C14 | 179.7 | 179.8 | 179.6 |
| C8−H6 | 1.082 | 1.082 | 1.081 | C1−C7−C8 | 126.2 | 125.6 | 125.3 | C9−C10−C11−O3 | 0.183 | 0.187 | 0.135 |
| C9−O4 | 1.255 | 1.242 | 1.260 | C7−C8−H6 | 120.7 | 120.7 | 119.9 | C10−C11−C12−H8 | -180.0 | -179.9 | -179.8 |
| C10−C11 | 1.426 | 1.421 | 1.422 | C7−C8−C9 | 121.6 | 121.1 | 121.3 | C11−C12−C13−O2 | -179.9 | -179.9 | -179.8 |
| C11−C12 | 1.393 | 1.389 | 1.390 | H6−C8−C9 | 117.8 | 118.1 | 118.7 | C10−C15−C14−H10 | 179.7 | 179.7 | 179.8 |
| C12−C13 | 1.399 | 1.396 | 1.399 | C8−C9−C10 | 115.4 | 115.0 | 115.0 | C12−C13−O2−H9 | 179.7 | 178.9 | 179.6 |
| C13−C14 | 1.402 | 1.397 | 1.395 | C8−C9−O4 | 122.7 | 122.7 | 122.6 | C10−C11−O3−H7 | -0.055 | -0.026 | -0.024 |
| C14−C15 | 1.391 | 1.388 | 1.393 | O4−C9−C10 | 121.9 | 122.2 | 122.3 | O1−C7−C8−C9 | -0.984 | -0.920 | -0.973 |
| C11−O3 | 1.340 | 1.334 | 1.352 | C9−C10−C15 | 120.1 | 120.1 | 119.9 | C9−C10−C15−O1 | -0.4495 | -0.415 | -0.461 |
| O3−H7 | 0.998 | 0.989 | 0.991 | C9−C10−C11 | 121.8 | 121.8 | 121.8 | C10−C15−O1−C7 | 0.701 | 0.690 | 0.701 |
| C12−H8 | 1.083 | 1.083 | 1.081 | C11−C10−C15 | 118.1 | 118.1 | 117.7 | C11−C12−C13−C14 | 0.057 | 0.120 | 0.124 |
| C13−O2 | 1.364 | 1.355 | 1.372 | C10−C15−O1 | 120.7 | 120.9 | 121.4 | C13−C14−C15−C10 | 0.013 | -0.034 | 0.040 |
| O2−H9 | 0.966 | 0.964 | 0.967 | C10−C15−C14 | 122.6 | 122.6 | 122.6 | C12−C13−C14−C15 | -0.061 | -0.125 | -0.055 |
| C14−H10 | 1.085 | 1.084 | 1.083 | C14−C15−O1 | 116.7 | 116.5 | 115.8 | C13−C14−C15−O1 | -179.8 | -179.8 | -179.6 |
|  |  |  |  | C15−O1−C7 | 120.8 | 120.5 | 119.2 | C14−C15−C10−C11 | 0.039 | 0.189 | 0.375 |
|  |  |  |  | C10−C11−C12 | 120.2 | 120.3 | 120.3 | C12−C13−C14−H10 | -179.8 | -179.9 | -179.8 |
|  |  |  |  | C10−C11−O3 | 120.0 | 120.3 | 120.6 |  |  |  |  |
|  |  |  |  | O3−C11−C12 | 119.8 | 119.4 | 119.0 |  |  |  |  |
|  |  |  |  | C11−O3−H7 | 106.8 | 107.7 | 106.4 |  |  |  |  |
|  |  |  |  | C11−C12−C13 | 119.4 | 119.3 | 119.5 |  |  |  |  |
|  |  |  |  | C11−C12−H8 | 120.3 | 120.3 | 120.2 |  |  |  |  |
|  |  |  |  | H8−C12−C13 | 120.3 | 120.3 | 120.2 |  |  |  |  |
|  |  |  |  | C12−C13−C14 | 122.0 | 122.1 | 121.8 |  |  |  |  |
|  |  |  |  | C12−C13−O2 | 116.5 | 116.4 | 116.1 |  |  |  |  |
|  |  |  |  | O2−C13−C14 | 121.5 | 121.5 | 122.1 |  |  |  |  |
|  |  |  |  | C13−O2−H9 | 110.3 | 110.5 | 109.4 |  |  |  |  |
|  |  |  |  | C13−C14−C15 | 117.6 | 117.6 | 117.6 |  |  |  |  |
|  |  |  |  | C13−C14−H10 | 122.1 | 122.4 | 122.4 |  |  |  |  |
|  |  |  |  | H10−C14−C15 | 120.2 | 120.1 | 119.8 |  |  |  |  |
